# Formation of Organic Fouling during Membrane Desalination: The Effect of Divalent Cations and the Use of an Online Visual Monitoring Method

**DOI:** 10.3390/membranes12121177

**Published:** 2022-11-23

**Authors:** Yaal Lester, Amit Hazut, Assaf Spanier

**Affiliations:** 1 Environmental Technologies, Department of Materials Engineering, Azrieli College of Engineering, Jerusalem 9103501, Israel; 2 Department of Software Engineering, Azrieli College of Engineering, Jerusalem 9103501, Israel

**Keywords:** reverse osmosis, fouling, divalent cations, online monitoring, image analysis

## Abstract

Reverse osmosis (RO) is the most popular technology for brackish, seawater and wastewater desalination. An important drawback of RO is membrane fouling, which reduces filtration effectiveness and increase the cost of produced water. This study addresses two important topics of membrane fouling: (i) the impact of different divalent ions on the formation of organic fouling and (ii) online monitoring and prediction of fouling formation. In the absence of divalent ions, dissolved organic matter had little effect on fouling formation, even at 3.5 mgC/L, in the upper range of groundwater concentration. Calcium, strontium and iron enhanced (organic) fouling formation, whereas barium had negligible effect. However, while iron affected fouling throughout the entire tested range (0–0.5 mg/L), calcium and strontium enhanced organic fouling only at high concentrations: more than 140 mg/L and 10 mg/L for calcium and strontium, respectively. An online system was developed for monitoring the formation of organic fouling, consisting of (i) an ex-situ RO cell with a transparent cover, (ii) a video camera continually monitoring the surface of the membrane and (iii) an algorithm which automatically identified changes in the color of the membrane caused by fouling, using a specially designed membrane spacer with colored reference dots. Changes in the color of the membrane surface were normalized to the reference colors, to eliminate all non-fouling related interference. The system was used to record and analyze changes in membrane color during numerous filtration tests. The data was successfully correlated to changes in specific flux (and subsequently to fouling formation rate) and can be applied to monitor and predict the formation of membrane fouling during desalination.

## 1. Introduction

Reverse osmosis (RO) desalination is a leading technology for the augmentation of potable water supply [1]. An important application of RO is brackish water desalination, which has experienced an exponential growth over the last 20 years, notably in arid regions of the world [2]. Brackish water typically refers to (ground)water with total dissolved solids (TDS) in the 1000–10,000 mg/L range (https://water.usgs.gov (accessed on 2 April 2022)). In the US, for example, over 77 percent of desalination plants in 2013 were fed by brackish groundwater, mostly in dry states like Florida, Texas, California and New Mexico [3]. In Israel is currently operating several brackish water desalination plants, producing up to 87 million m^3^/year, approximately 12% of total desalinated water (Israel Water Authority).

One of the main challenges in RO desalination is membrane fouling, caused by the deposition of suspended or dissolved material on membrane surface. Fouling reduces permeate flux, increases energy demand, forces frequent membrane cleaning, reduces membrane lifetime and in general increases RO operational cost [4,5,6]. Membrane foulants in brackish groundwater mainly comprise of inorganic ions, suspended and colloidal solids and low levels of natural organic matter—NOM [7]. The relative contribution of each component may vary significantly, depending on the groundwater source and RO pretreatment. 

Important ions, which may form insoluble salt precipitates (scaling), include calcium (Ca^2+^), magnesium (Mg^2+^), barium (Ba^2+^), strontium (Sr^2+^), sulphate and carbonate [3]. Karime et al. [8] showed that insoluble salts such as CaSiO_3_, Fe_3_O_4_, AlPO_4_ and CaSO_4_ were key membrane foulant agents in a brackish water desalination plant, working since 1999 in Tunisia. On the other hand, Yang et al. [7] showed that organic substances are dominant foulants, at least in the first stage of brackish water desalination. Ruiz-García et al. [9] autopsied membrane elements of a full-scale brackish groundwater desalination plant, after 11 years of operation, and found that fouling consisted of a biofilm (mainly in the first elements of the RO system) and inorganic foulants of calcium carbonate and aluminosilicates. Reducing scaling can be done through the application of anti-scalants, which improve membrane recovery but increase treatment cost [10,11,12,13,14].

A different fouling formation mechanism which involves inorganic ions (in addition to scaling) is the synergistic effect of cations and organic matter. Interaction of NOM with (divalent) cations may result in metal-NOM complexation and/or the formation of “bridges” between carboxylic groups of organic molecules, which promotes the formation of membrane fouling [15]. Lee et al. [16] found that calcium ions significantly enhanced membrane fouling during RO treatment of alginate, a model polysaccharide in secondary wastewater effluent. The increase in fouling was attributed to the formation of calcium alginate complexation and crosslinking (bridging). Magnesium ions on the other hand had little effect on fouling formation under similar conditions. Zaho et al. [17] showed that RO membrane fouling, caused by humic acid, was largely aggravated in the presence of calcium, causing a significant flux decline. Their analysis revealed that addition of calcium increased particles size of humic acid. A similar phenomenon was demonstrated by other researchers for calcium and magnesium [18,19,20], yet no study has ever examined the impact of other relevant cations such as strontium and barium, despite their proven bridging potential and frequent presence in brackish groundwater [21]. Strontium was detected in the vast majority of US groundwater aquifers at concentrations typically below 1 mg/L, but levels higher than 4 mg/L (and even peaks of >30 mg/L) were also observed, principally in aquifers with Sr-bearing rocks [22]. Barium also naturally occurs in many groundwaters, at levels typically lower than 1 mg/L, but may reach as high as 20 mg/L in specific locations [23].

Another aspect of membrane fouling, which has attracted growing attention over the last years, is online monitoring, and the prediction of fouling formation rate [24,25,26]. Standard full-scale methods involve the indirect measurement of trans-membrane pressure or flux decline, and the setting of a threshold for membrane cleaning [4]. However, these parameters are relatively insensitive, and do not provide any future prediction. Other methods tested at small scale include the use of magnetic resonance measurements [27], electrical impedance spectroscopy [28] and ATP growth potential [29]. Recently, Cohen and his group [30] proposed a different approach, using an on- line ex situ plate-and-frame membrane cell, connected on a side stream of the RO feed and operating under similar conditions. The cover of the cell is transparent, and the exposed membrane surface is continuously monitored using a video camera and a surface image analyzer. This system was successfully applied for detecting mineral scaling formation and for the auto-initiation of a feed flow reversal system. However, since the system is based on the identification of distinctive geometrical shapes (particles), its application is limited for detecting mineral scaling. Organic or microbial fouling will mostly lead to changes in membrane color, rather than formation of particles. To widen the use of this approach, a method should be developed to identify changes in membrane color caused by (organic) fouling and correlate these to changes in hydraulic parameters such as flux and pressure. 

The goals of this study were to (i) determine the effect of different divalent cations on the formation of organic fouling during brackish water desalination and (ii) develop an online method for monitoring the formation of organic membrane fouling. This method makes use of an on-line plate-and-frame RO cell with a specially designed spacer, and an algorithm, which automatically identify changes in the color of the membrane surface caused by fouling. 

## 2. Materials and Methods

### 2.1. Reagents and Chemicals

Suwannee River fulvic acid (SRFA) was obtained from the International Humic Substances Society (www.ihss.gatech.edu (accessed on 23 April 2022)). NaCl, SrCl_2_, BrCl_2_, CaCl_2_ and FeCl_3_ were obtained from Sigma-Aldrich and were at least analytical grade (>98%). All chemicals were used as received.

### 2.2. Setup of the Lab-Scale RO System

**Figure 1:**
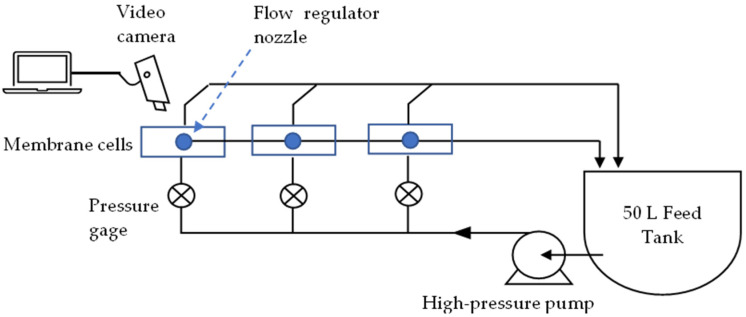
Schematic of the lab-scale crossflow RO system.

Experiments were carried out using a lab-scale crossflow RO system, consisting of three membrane cell units connected in parallel (CF-16, Sterlitech). The cells are 12.7 × 10 × 8.3 cm in size, with an active membrane area of 20.6 cm^2^ (each). They are fed by a high-pressure pump connected to a 50 L temperature-controlled feed container (Figure 1). To maintain a constant feedwater flowrate, special nozzles were installed on the brine-side of the cells and feed pressure was continuously monitored using pressure gauges. Flowrate and conductivity of permeates and brines were measured and recorded four times a day. Membranes used for this study were brackish water thin film composite polyamides (SUEZ, AG, PA-TFC, Table S1 in Supplementary Materials).

For real-time monitoring of the membrane surface (feed-side), the upper part of one of the cells was replaced by a transparent acrylic block. A video camera attached to a monoscope was placed above the cell (HAYEAR Full HD 34MP Industrial Electronic Digital Video Microscope Camera) and focused on a small portion of the membrane surface. The camera was connected to a designated software (Hayer version ×64), allowing the consecutive recording of 72 h videos. Constant lightning was supplied from a ring of LED lamps connected to the camera.

### 2.3. Fouling Experiments

Experiments were carried out using synthetic (lab-made) brackish water, based on the analysis of feed water from Maagan Michael plant, Israel’s largest brackish water desalination facility (detailed analysis in Supporting Information). In a typical experiment, the feedwater tank was first filled with tap water from the Environmental Technologies laboratory (Azrieli College Jerusalem). NaCl was then added from a stock solution to a final concentration of 3000 mg/L (conductivity of approximately 6400 μS/cm). Other constituents were then added from filtered stock solutions (fulvic acid, CaCl_2_, BaCl_2_, SrCl_2_, FeCl_3_), depending on the experiment, and relevant quality parameters were measured: alkalinity, hardness, pH, DOC, conductivity. Membrane coupons were soaked in saline water for 12 h prior to installation. Water was circulated through the membrane cells at a constant flowrate of approximately 1 L/min (for each cell) and feed pressure of 18 bar. Sampling and image recording began after two hours of equilibration, for a total duration of 72 h. 

### 2.4. Image Processing and Analysis

**Figure 2:**
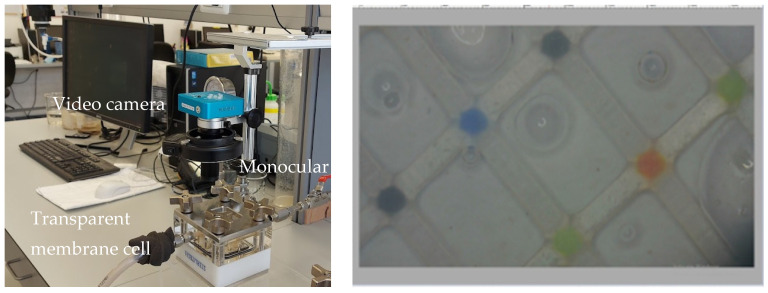
The video camera mounted above a transparent cell (**left**) and a screenshot of the recorded video, with the specially designed membrane spacer (**right**).

The real-time monitoring system consisted of a video camera mounted above a transparent cell, recording membrane surface and the accumulation of fouling (Figure 2 left). To differentiate between color changes of membrane due to fouling and changes resulting from lightning or other artifacts, a specially designed spacer was installed in the transparent cell, that featured dots of different colors according to a standard color chart: red, blue, green and black (Figure 2 right). An image analysis algorithm was developed to identify the colored dots automatically during filtration and normalize the membrane color to the pre-defined colors (the algorithm is further detailed in Section 3.2). Changes in the normalized color of the membrane during filtration were further correlated to changes in hydraulic parameters of the system. 

### 2.5. Analytical Methods

Conductivity was measured with a portable conductivity meter from Hach (HQ30D). Ions in the water were quantified by an ECO Ion Chromatograph (Metrohm, Switzerland), using EPA method 300.00. Dissolved organic matter (DOM) was measured using a TOC analyzer (Torch, TeledyneTekmar, OH, USA) after filtering the samples at 0.45 mm (APHA, method 5310B). Metals were quantified by Inductively Coupled Plasma Optical 143 Emission Spectrometry (ICP-OES, Spectro Genesis, Kleve, Germany).

## 3. Results and Discussion

Results are presented in two parts. The first focuses on the impact of DOM and divalent ions on the formation rate of organic fouling; whereas the second demonstrates the online method for monitoring the formation of organic membrane fouling. 

### 3.1. Formation of Membrane Fouling

#### 3.1.1. The Effect of DOM Concentration on Fouling Formation 

**Figure 3:**
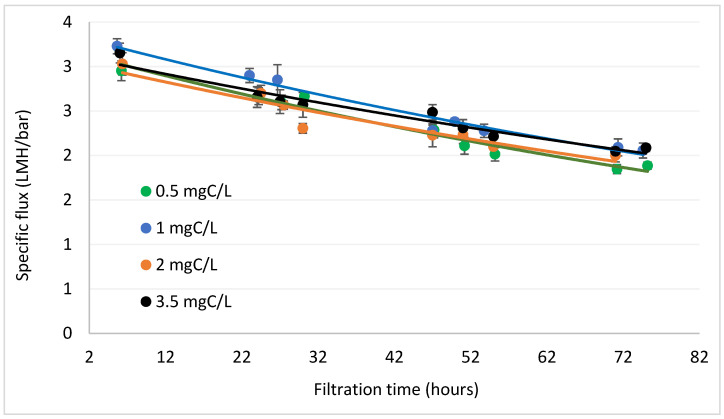
Decrease in specific flux for different concentrations of organic matter.

Experiments in this section were carried out with tap water, spiked with NaCl and different concentrations of fulvic acid (0.5–3.5 mgC/L, in the upper range of groundwater) [31,32]. Background quality parameters of the water were: Alkalinity 155 ± 10 mg/L as CaCO_3_, Ca 42 ± 2, pH 8.3 and conductivity of 6210 ± 120 μS/cm. Formation rate of fouling was monitored over a period of 72 h, using specific flux as an indicator, flux divided by transmembrane pressure (Flux/TMP, LMH/bar) [33]. Increasing the concentration of organic matter had little effect on the rate of decrease of the specific flux: the slope of the exponential regression lines in Figure 3. This implies that, at the tested concentration range (≤3.5 mgC/L) and specific water chemistry, DOM makes little contribution to membrane fouling. 

Literature data regarding the effect on DOM concentration on the formation of organic fouling is typically site-specific, and depends on the chemistry of the feedwater, hydraulic parameters and pretreatment. Miyoshi et al. [34], for example, did not find any correlation between TOC or DOC concentration in seawater and RO fouling formation rate (at TOC range ≤ 4 mgC/L). On the other hand, Koyuncu et al. [35] showed that reducing the concentration of DOC in surface water from 2.94 to 1.19 mg/L improved the rate of flux decrease during desalination by almost 30%. A possible explanation for these different effects could be the presence of calcium in the water. In the case of Koyuncu [35], calcium concentration was in the range of 79–106 mg/L (no calcium concentration was given by Miyoshi et al. [34]. The level of calcium in the water decreased during desalination, in parallel to the decrease in TOC. Koyuncu hence concluded that the impact of organic matter on membrane fouling at low concentration relates to its interaction with calcium. In our experiments the concentration of calcium was relatively low: 40 mg/L, which may explain the marginal effect of organic matter on fouling formation. This hypothesis is validated in the following section.

#### 3.1.2. The Combined Effect of DOM and Calcium on Fouling Formation

**Figure 4:**
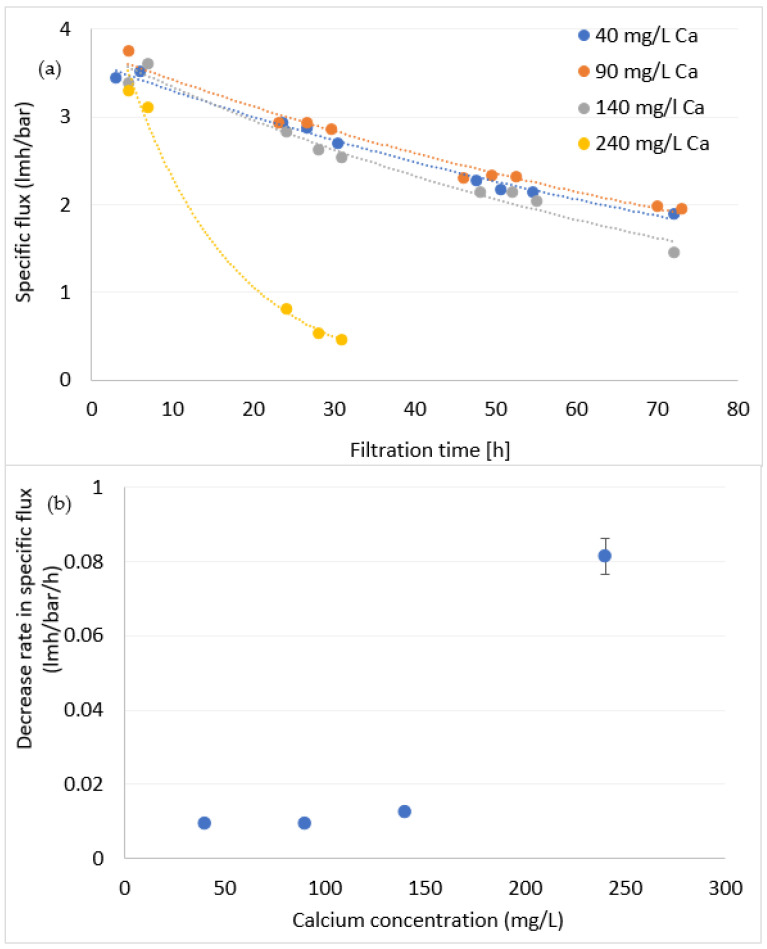
Specific flux vs. time (**a**) and the exponential decrease rate in specific flux for different concentrations of calcium (**b**). DOC = 3.5 mgC/L.

In this section, DOM concentration was kept constant at 3.5 mgC/L and calcium concentration was increased up to 240 mg/L. Figure 4a illustrates the changes in specific flux over time for the different calcium concentrations and Figure 4b summarizes the decrease rates. Increasing calcium concentration up to 140 mg/L had little effect on the decrease rate of specific flux, hence on the formation rate of fouling. On the other hand, at higher concentration of 240 mg/L, calcium sharply increased fouling formation rate. This indicates Ca–DOM interaction as the dominant mechanism for fouling formation under these conditions. Previous work also observed a critical concentration of Ca, where DOM becomes insoluble and sharply affects membrane flux [36]. This phenomenon, typically noticed around 100 mg/L (depend on DOM concentration), was attributed to charge neutralization and subsequent precipitation of organic macromolecules [37]. Scaling of CaCO_3_ is less plausible, as the water’s CaCO_3_ precipitation potential was calculated at −14.3 (using STASOFT 5). 

Similar results were found in previous studies [18,20,36,38], which largely validate the assumption in Section 3.1.1: the impact of organic matter at low levels on membrane fouling depends on the concentration of calcium, and possibly other divalent cations, in the water.

#### 3.1.3. The Effect of Strontium, Barium and Iron on Formation of Organic Fouling 

Unlike the well-documented impact of calcium, the effect of strontium and barium on organic fouling is unknown. The effect of iron was mostly documented in the context of flocculation, where it is added to increases in small organic colloids and produced removable flocs. This phenomenon, however, does not always take place in natural water, especially under conditions of dissolved organic matter and low levels of iron. 

**Figure 5:**
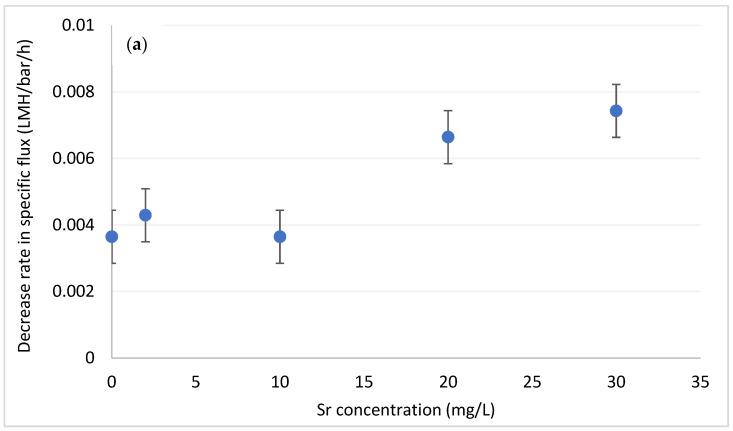

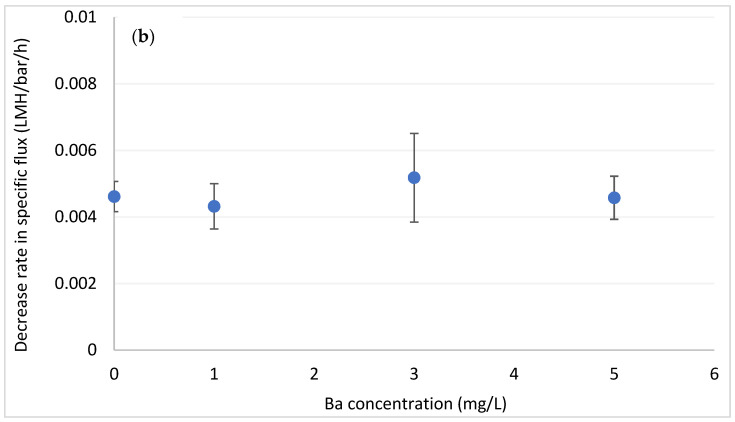
Decrease in specific flux for different concentrations of (**a**) strontium and (**b**) barium. DOC = 3.5 mgC/L.

Figure 5 presents the rate constants for the decrease in specific flux as function of strontium and barium concentrations, at levels up to 30 mg/L and 5 mg/L, respectively, (in the upper range of groundwater concentration) [23,39]. Interestingly, the two divalent ions exhibit different effects on fouling formation. Strontium up to 10 mg/L did not affect fouling, while at higher concentrations it promoted fouling formation. This effect is similar to the one observed for calcium (though to a smaller extent), and is likely the result of strontium–DOM interaction and the increase in the size of organic flocs. Barium, on the other hand, had marginal effect on fouling formation throughout the tested range. To verify that the negligible effect of barium was not concentration-dependent, we extended its tested range up to 30 mg/L (similar to Sr^2+^). Even at this high range, barium did not promote fouling formation (results not shown), which suggest that its tendency to interact with DOM is lower than that of strontium (and calcium). It should be emphasized that sulphate was not detected in the water at any point, hence fouling could not be attributed to the insoluble SrSO_4_ or BaSO_4_. 

An explanation for the observed trends may be drawn from the work of Rios-Carvajal [21], which investigated (at nanometer scale) the interactions of different carboxylate functionalities in organic molecules with dissolved divalent cations. They found that carboxylates and dicarboxylates interact differently with different ions. For example, adhesion force of carboxylate with divalent ions increased in the order of Ba^2+^ > Ca^2+^ > Sr^2+^. On the other hand, in systems of dicarboxylate, interaction was weakest for barium (and stronger for strontium and calcium). The researchers concluded that the strong interaction of strontium with dicarboxylate functional groups resulted from a chelation bidentate interaction, rather than an intermolecular bridging. Our results therefore suggest that the applied DOM (fulvic acid) contained more dicarboxylate functional moieties than carboxylate, thus encouraging DOM interaction with calcium and strontium and discouraging the effect of barium.

**Figure 6:**
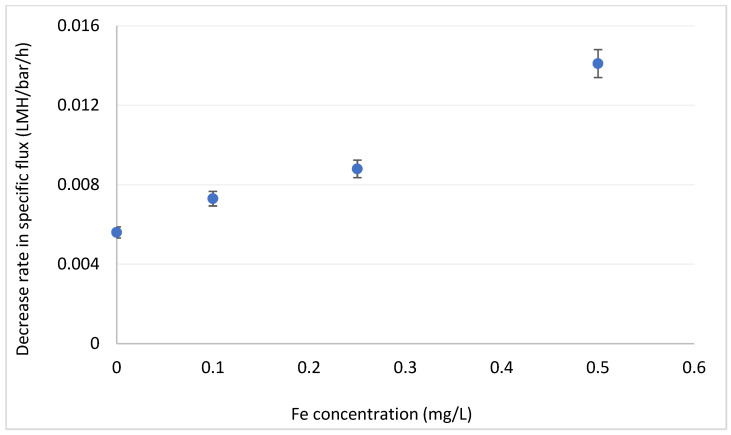
Decrease in specific flux for different concentrations of iron. DOC = 3.5 mgC/L.

Iron (added as FeCl_3_) enhances fouling formation even at the lowest concentration tested (Figure 6). These results are highly important, since iron is omnipresent in many brackish water sources in Israel and elsewhere [40] and its effect on organic fouling should be taken into consideration. Control tests of with iron (0.1 and 0.5 mg/L) but without DOM showed that iron alone have little effect on fouling formation rate. 

### 3.2. Online Visual Monitoring of Fouling Formation

The goal in this section was to develop an online monitoring system for predicting and alerting rapid fouling formation. The system combines online video recording of a membrane section and a video analyzing algorithm. Analysis consisted of two steps: (1) auto-identification algorithm of the reference-colored dots on the spacer and (2) scalar value normalization based on the given reference-colored dots.

**Figure 7:**
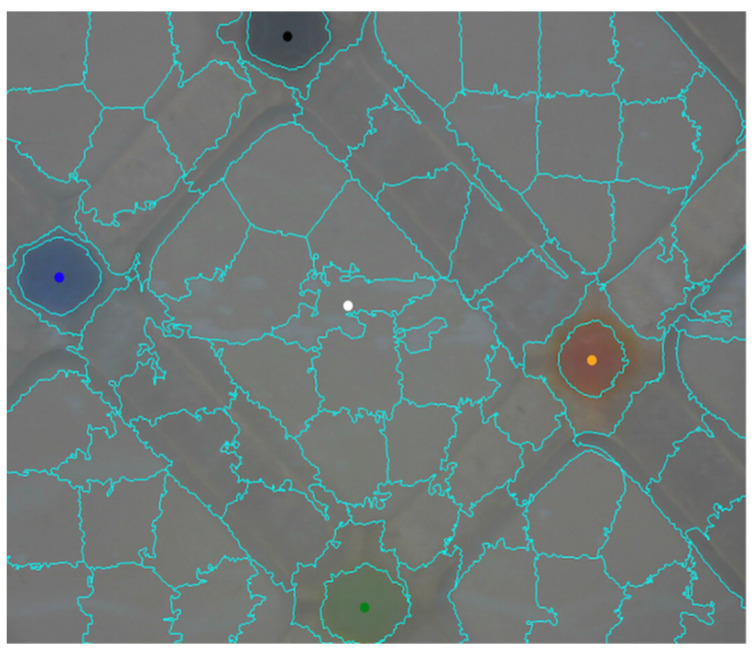
The super-pixels map, including the automatic placement of the colored dots: a black dot in the center of a black super-pixel, a blue dot in the center of a blue super-pixel etc. A super-pixel marked in white at the center indicates the area used for fouling analysis.

Auto-identification of the colored dots and their fixation for the entire duration of the experiment was not straightforward, since the coordinates and the color of the dots varies continually, due to fouling accumulation and minor movements of the flow-cell. For that purpose, the SLIC (Simple Linear Iterative Clustering) algorithm was used on each image of the video to create compacts, nearly uniforms super-pixels by clustering image pixels in a color space of five dimensions (three color dimension and two space dimensions), resulting in a map of the super-pixels, as can be seen in Figure 7. Subsequently, the image processing method identified a central area of the membrane (marked with white dot in Figure 7), for analysis of membrane color change during filtration. Note that the first image of the video was utilized for locating the reference-colored dots, since the contrast between the dotes and the membrane was relatively high (no fouling accumulation yet). The dots’ location was further used during the rest of the experiment, with continuous adjustments between successive frames, to track the exact position of the colored and central super-pixels. Details of the SLIC algorithm is provided in the Supporting Information.

The next step was the analysis of changes in membrane color attributed to fouling. For that, colors of all super-pixels were fragmented into their red-green-blue components, and the specific component in the central super-pixel was divided by its counterparts in the reference colored super-pixels. This was done continually during the entire duration of the experiment to eliminate color changes due to lightning and other artifacts. The three components underwent gray transformation to obtain a single representative value. The green super-pixel was eventually selected for comparison with the hydraulic data, based on its superior stability and visibility throughout the experiment. 

**Figure 8:**
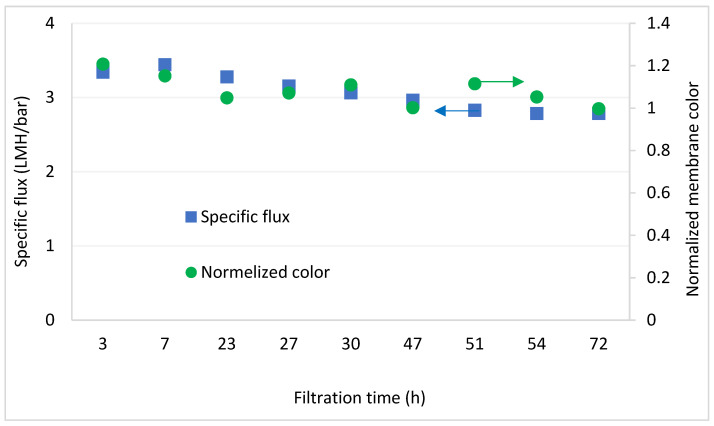
Decrease in specific flux and membrane color normalized to the green reference dot, during an entire filtration experiment from Section 3.1.2 (Ca = 90 mg/L).

Changes in normalized color of the membrane vs. time were plotted in parallel to changes in specific flux for each experiment, as exemplified in Figure 8. Correlation between the hydraulic and visual parameter was tested using Equation (1). All experiments exhibited correlation higher than 0.8, with an average of 0.89, indicating high correlation between the two parameters.

(1) $$Correl\left(X,Y\right)=\frac{\sum (x-\bar{x)\left(y-\overset{=}{y}\right)}}{\sqrt{\sum {\left(x-\bar{x}\right)}^{2}\sum {\left(y-\bar{y}\right)}^{2}}}$$

Here, $\bar{x}$ and $\bar{y}\;$ are the sample means

**Figure 9:**
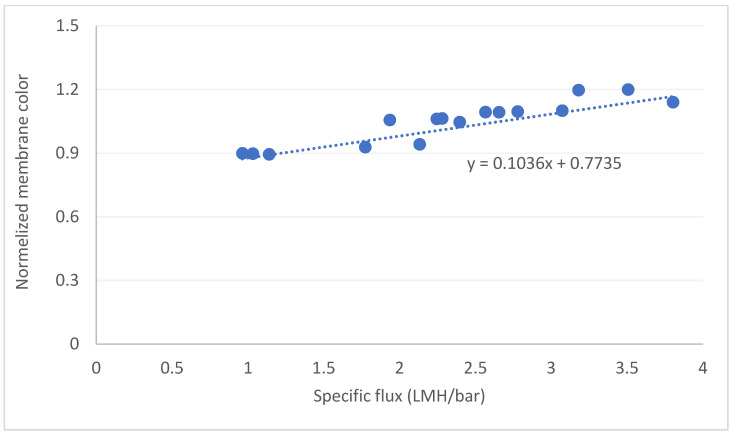
Example correlations between the normalized membrane color and specific flux.

In the final stage, specific flux was plotted against the relative color intensity (Figure 9 as an example), and the linear regression was calculated for all experiments. Interestingly, all experiments involving DOM at ≥ 2 mgC/L exhibited correlation factors (the slop of the linear regression) in the narrow range of 0.09–0.11, whereas experiments with lower DOM concentrations showed high variability. These results suggest that (i) the main foulant in our system is organic matter and that (ii) the normalized color intensity can be applied for monitoring and predicting the formation of fouling in a variety of water quality. In other words, by monitoring the changes in normalized membrane color and applying a pre-measured correlation factor, we can predict the decrease in specific flux and the formation rate of membrane fouling, which can be used, for example, for coordinating a membrane backwash cycle.

## 4. Conclusions

Membrane fouling is a major drawback of RO desalination, affecting the operation of seawater and brackish water desalination facilities. This study addresses two important points in membrane fouling: (i) the impact of different divalent ions on organic fouling and (ii) the use of online visual monitoring and predicting of fouling formation. The following conclusions could be drawn from the experimental work:

- When divalent ions are at low concentrations, organic fouling is insignificant, even when DOM is at the upper range of groundwater concentrations (3.5 mgC/L).
- Of the divalent ions tested, calcium, iron and strontium were shown to enhance the formation of organic fouling, whereas barium did not affect fouling formation, likely due to its low affinity to dicarboxylate moieties in the DOM.
- Visual online monitoring of fouling formation was successful, using reference color-dots positioned on the spacer. In this system, membrane color change was continuously recorded and normalized to the reference color dots, thus eliminating interferences from lightning and other environmental factors.
- The normalized color showed high correlation to the decrease in specific flux and can be used in the future to monitor and predict fouling formation during membrane desalination at full-scale.

## Data Availability

Raw data can be obtained from the author on request.

## Supplementary Materials

The following supporting information can be downloaded at: https://www.mdpi.com/article/10.3390/membranes12121177/s1, Table S1. Specifications of the RO membranes used in the study; Table S2. Quality parameters of the brackish water from Maagan Michael desalination facility; Text S1: The pseudocode of the SLIC algorithm, with more details below.

## References

[1] Greenlee L.F., Lawler D.F., Freeman B.D., Marrot B., Moulin P. (2009). Reverse osmosis desalination: Water sources, technology, and today’s challenges. Water Res..

[2] Honarparvar S., Zhang X., Chen T., Alborzi A., Afroz K., Reible D. (2021). Frontiers of membrane desalination processes for brackish water treatment: A review. Membranes.

[3] Voutchkov N. (2013). Desalination Engineering Planning and Design.

[4] Jiang S., Li Y., Ladewig B.P. (2017). A review of reverse osmosis membrane fouling and control strategies. Sci. Total Environ..

[5] Goh P.S., Lau W.J., Othman M.H.D., Ismail A.F. (2018). Membrane fouling in desalination and its mitigation strategies. Desalination.

[6] Tong T., Wallace A.F., Zhao S., Wang Z. (2019). Mineral scaling in membrane desalination: Mechanisms, mitigation strategies, and feasibility of scaling-resistant membranes. J. Membr. Sci..

[7] Yang H.L., Huang C., Pan J.R. (2008). Characteristics of RO foulants in a brackish water desalination plant. Desalination.

[8] Karime M., Bouguecha S., Hamrouni B. (2008). RO membrane autopsy of Zarzis brackish water desalination plant. Desalination.

[9] Ruiz-garcía A., Melián-martel N., Mena V. (2018). Fouling characterization of RO membranes after 11 years of operation in a brackish water desalination plant. Desalination.

[10] Jiang W., Xu X., Johnson D., Lin L., Wang H., Xu P. (2022). Effectiveness and mechanisms of electromagnetic field on reverse osmosis membrane scaling control during brackish groundwater desalination. Sep. Purif. Technol..

[11] Ruiz-García A., Nuez I., Carrascosa-Chisvert M.D., Santana J.J. (2020). Simulations of BWRO systems under different feedwater characteristics. Analysis of operation windows and optimal operating points. Desalination.

[12] Alshami A., Taylor T., Ismail N., Buelke C., Schultz L. (2021). RO system scaling with focus on the concentrate line: Current challenges and potential solutions. Desalination.

[13] Mangal M.N., Salinas-Rodriguez S.G., Dusseldorp J., Kemperman A.J.B., Schippers J.C., Kennedy M.D., van der Meer W.G.J. (2021). Effectiveness of antiscalants in preventing calcium phosphate scaling in reverse osmosis applications. J. Membr. Sci..

[14] Karanasiou A., Karabelas A.J., Mitrouli S.T. (2021). Incipient membrane scaling in the presence of polysaccharides during reverse osmosis desalination in spacer-filled channels. Desalination.

[15] Van Geluwe S., Braeken L., Van der Bruggen B. (2011). Ozone oxidation for the alleviation of membrane fouling by natural organic matter: A review. Water Res..

[16] Lee S., Ang W.S., Elimelech M. (2006). Fouling of reverse osmosis membranes by hydrophilic organic matter: Implications for water reuse. Desalination.

[17] Zhao X., Wu Y., Zhang X., Tong X., Yu T., Wang Y., Ikuno N., Ishii K., Hu H. (2019). Ozonation as an efficient pretreatment method to alleviate reverse osmosis membrane fouling caused by complexes of humic acid and calcium ion. Front. Environ. Sci. Eng..

[18] Wang L.F., He D.Q., Chen W., Yu H.Q. (2015). Probing the roles of Ca^2+^ and Mg^2+^ in humic acids-induced ultrafiltration membrane fouling using an integrated approach. Water Res..

[19] Li Q., Elimelech M. (2004). Natural organic matter fouling and chemical cleaning of nanofiltration membranes. Water Sci. Technol. Water Supply.

[20] Al-Amoudi A.S. (2010). Factors affecting natural organic matter (NOM) and scaling fouling in NF membranes: A review. Desalination.

[21] Rios-Carvajal T., Bovet N., Bechgaard K., Stipp S.L.S., Hassenkam T. (2019). Effect of divalent cations on the interaction of carboxylate self-assembled monolayers. Langmuir.

[22] Musgrove M.L. (2021). The occurrence and distribution of strontium in U.S. groundwater. Appl. Geochemistry.

[23] Moore R.B., Staubitz W.W. (1984). Distribution and Source of Barium in Ground Water at Cattaraugus Indian Reservation, Southwestern New York.

[24] Weinrich L., Haas C.N., Lechevallier M.W. (2013). Recent advances in measuring and modeling reverse osmosis membrane fouling in seawater desalination: A review. J. Water Reuse Desalin..

[25] Sim L.N., Chong T.H., Taheri A.H., Sim S.T.V., Lai L., Krantz W.B., Fane A.G. (2018). A review of fouling indices and monitoring techniques for reverse osmosis. Desalination.

[26] Gu H., Rahardianto A., Gao L.X., Caro X.P., Giralt J., Rallo R., Christofides P.D., Cohen Y. (2018). Fouling indicators for field monitoring the effectiveness of operational strategies of ultrafiltration as pretreatment for seawater desalination. Desalination.

[27] Bristow N.W., Vogt S.J., Bucs S.S., Vrouwenvelder J.S., Johns M.L., Fridjonsson E.O. (2021). Novel magnetic resonance measurements of fouling in operating spiral wound reverse osmosis membrane modules. Water Res..

[28] Ho J.S., Low J.H., Sim L.N., Webster R.D., Rice S.A., Fane A.G., Coster H.G.L. (2016). In-situ monitoring of biofouling on reverse osmosis membranes: Detection and mechanistic study using electrical impedance spectroscopy. J. Membr. Sci..

[29] Abushaban A., Salinas-Rodriguez S.G., Kapala M., Pastorelli D., Schippers J.C., Mondal S., Goueli S., Kennedy M.D. (2020). Monitoring biofouling potential using ATP-based bacterial growth potential in SWRO pre-treatment of a full-scale plant. Membranes.

[30] Uchymiak M., Bartman A.R., Daltrophe N., Weissman M., Gilron J., Christofides P.D., Kaiser W.J., Cohen Y. (2009). Brackish water reverse osmosis ( BWRO ) operation in feed flow reversal mode using an ex situ scale observation detector (EXSOD). J. Membr. Sci..

[31] Perdue E.M., Ritchie J.D. (2003). Dissolved organic matter in freshwaters. Treatise Geochem..

[32] Rutlidge H., McDonough L.K., Oudone P., Andersen M.S., Meredith K., Chinu K., Peterson M., Baker A. (2021). Characterisation of groundwater dissolved organic matter using LC[sbnd]OCD: Implications for water treatment. Water Res..

[33] Brehant A., Bonnelye V., Perez M. (2003). Assessment of ultrafiltration as a pretreatment of reverse osmosis membranes for surface seawater desalination. Water Sci. Technol. Water Supply.

[34] Miyoshi T., Hayashi M., Shimamura K., Matsuyama H. (2016). Important fractions of organic matter causing fouling of seawater reverse osmosis (SWRO) membranes. Desalination.

[35] Koyuncu I., Wiesner M.R., Bele C., Coriton G., Djafer M., Cavard J. (2006). Bench-scale assessment of pretreatment to reduce fouling of salt-rejecting membranes. Desalination.

[36] Hong S., Elimelech M. (1997). Chemical and physical aspects of natural organic matter (NOM) fouling of nanofiltration membranes. J. Membr. Sci..

[37] Amirbahman A., Olson T.M. (1995). Deposition kinetics of humic matter-coated hematite in porous media in the presence of Ca^2+^. Colloids Surf. A Physicochem. Eng. Asp..

[38] Li Q., Elimelech M. (2004). Organic fouling and chemical cleaning of nanofiltration membranes: Measurements and mechanisms. Environ. Sci. Technol..

[39] Tedd K., Coxon C., Misstear B., Daly D., Craig M., Mannix A., Hunter Williams T. (2017). Assessing and Developing Natural Background Levels for Chemical Parameters in Irish Groundwater Identifying Pressures Developing Solutions.

[40] Rosenthal E., Weinberger G., Berkowitz B., Flexer A., Kronfeld J. (1992). The Nubian Sandstone aquifer in the central and northern Negev, Israel: Delineation of the hydrogeological model under conditions of scarce data. J. Hydrol..

