# Supplementary material for: Formation of Organic Fouling during Membrane Desalination: The Effect of Divalent Cations and the Use of an Online Visual Monitoring Method
Source: Membranes (Basel). 2022 Nov 23;12(12):1177. doi: 10.3390/membranes12121177 (PMC9783477; doi:10.3390/membranes12121177)
Supplement: Supplementary file 1 [file membranes-12-01177-s001.zip › membranes-2019368-supplementary.pdf]

Supporting Information

Formation of organic fouling during membrane desalination: The effect of divalent cations and the use of online visual monitoring method

Table S1. Specifications of the RO membranes used in the study

Manufacturer	Suez (GE)
Type	Flat sheet Polyamide-TFC
Package	300 × 300 mm
Feed	Brackish Water
pH range	1–11
Flux (lmh)/bar	(44)/15.5
Rejection	95.5%
Pore size	N/A
Spacer	Medium Foulant, PP, 47 mil

*Data provided by the supplier: https://www.sterlitech.com

Table S2. Quality parameters of the brackish water from Maagan Michael desalination facility

	Average concentration mg/L	
B	0.39	± 0.01
Ba	1.9	±0.44
Ca	202.7	±0.11
Fe	0.2	±0.03
K	29.6	±3.4
Mg	165.1	±0.8
Mn	0.016	±0.001
Mo	0.017	±0.009
Na	900.6	±5.8
Ni	0.009	±0.001
P	0.064	±0.004
Sr	1.8554	±0.0114

DOC	2.1	±0.2
Alkalinity as $CaCO_3$	212	±25
Conductivity (μS/cm)	5870	±112

Text S1: The pseudocode of the SLIC algorithm, with more details below.

SLIC SuperPixel algo.

1. Initialized cluster center $c_k = [l_k, a_k, b_k, x_k, y_k]^T$ sampling pixels at regular grid steps S
2. For each pixel, set label ($l(i) = -1$)and the distance $d(i) = \infty$
3. Loop
4.   For (each cluster center c_k)
5.   For (each pixel i in $2S \cdot 2S$ region around C_k)
6.   Computer the distance D between $C_k$and i
7.   If (D<d(i))
8.   d(i) = D
9.   l(i) = k
10.   end if
11.   end for
12.   end for
13.   Update cluster centers
14.   Compute residual error E
15. Until E < threshold

- Line1: Initialization step, where k initial cluster centers in the CIELAB color space and location x,y. The cluster center are sample along equally spaced a grid with $S = \sqrt{imageSize/k}$
- Line 2: Initialization all labels $l(i)$ to -1 (i.e. no label) and the distance $d(i)$ to infinity.
- Line 5-10: The assignment step, each pixel i is associated with the nearest cluster center in a bounded region $2S \cdot 2S$. This is the speeding up step, because limiting the size of the search region significantly reduces the number of distance calculations.
- Line 14: The process ends when the residual error E is below some fixed threshold

The distance D is defined as follows:

$$D = \sqrt{{d_c}^2 + (d_s/S)^2 \cdot m^2} \qquad \text{(S1)}$$

The distance D normalize color proximity and spatial proximity by their respective maximum distances.
